# 表皮生长因子酪氨酸激酶抑制剂一线治疗突变阳性晚期非小细胞肺癌的临床疗效预测因素分析

**DOI:** 10.3779/j.issn.1009-3419.2019.02.04

**Published:** 2019-02-20

**Authors:** 闽江 陈, 燕 徐, 静 赵, 巍 钟, 孟昭 王

**Affiliations:** 100730 北京，中国医学科学院，北京协和医学院，北京协和医院呼吸内科 Department of Respiratory Medicine, Peking Union Medical College Hospital, Chinese Academy of Medical Sciences and Peking Union Medical College, Beijing 100730, China

**Keywords:** 肺肿瘤, 预测因素, 表皮生长因子受体酪氨酸激酶抑制剂, Lung neoplasms, Predictive factor, Epidermal growth factor receptor tyrosine kinase inhibitors

## Abstract

**背景与目的:**

一线应用表皮生长因子-酪氨酸激酶抑制剂（epidermal growth factor receptor tyrosine kinase inhibitors, EGFR-TKIs）治疗具有*EGFR*基因突变的晚期非小细胞肺癌（non-small cell lung cancer, NSCLC）疗效显著，但患者的无进展生存时间（progression free survival, PFS）可有较大差异。既往研究表明一些临床因素可能与疗效相关，本研究旨在探讨影响EGFR-TKI疗效的临床预测因素。

**方法:**

收集203例存在*EGFR*基因敏感突变且一线接受EGFR-TKI治疗的晚期NSCLC患者的人口学及临床资料并进行回顾性分析。

**结果:**

截至随访结束时203例患者中共有139例发生病情进展，63例死亡。中位随访时间为21.1个月，中位PFS为14.3个月。接受治疗患者疾病部分缓解（partial response, PR）127例（66.1%），疾病稳定（stable disease, SD）55例（28.6%）。与PFS相关的单因素分析结果显示，ECOG评分≥2分（5.1个月 *vs* 16个月，*P*=0.033）、最佳疗效为SD（9.5个月 *vs* 17.9个月，*P*=0.030）、合并胸腔外远处转移（11.7个月 *vs* 27.5个月，*P*=0.004）、肝转移（4.1个月 *vs* 16.0个月，*P*=0.000）、骨转移（13.3个月 *vs* 21.5个月，*P*=0.027）和同时并发肺栓塞（5.5个月 *vs* 16.6个月，*P*=0.005）的患者PFS明显缩短。多因素*Cox*回归结果显示合并肝转移（HR=1.694, 95%CI: 1.146-5.756, *P*=0.022）、最佳治疗反应仅达到SD（HR=1.825, 95%CI: 1.107-3.008, *P*=0.018）是独立的疗效预测因素。

**结论:**

对于*EGFR*突变阳性的晚期NSCLC患者，一线应用EGFR-TKI治疗效果良好。治疗的最佳疗效以及基线肝转移是PFS的独立临床预测因素。

## Abstract (en)

**Background and objective:**

Epidermal growth factor receptor tyrosine kinase inhibitors (EGFR-TKIs) have demonstrated some dramatic efficacy in advanced non-small-cell lung cancer (NSCLC) patients with activating *EGFR* mutation. However, progression-free survivals (PFS) among those patients who were treated with first line EGFR TKIs were inconsistent. The aim of this study is to explore the association of clinical prognostic factors with EGFR-TKI efficacy in advanced NSCLC patients.

**Methods:**

The demographic and clinical characteristics of 203 patients with activating *EGFR* mutation treated with first generation TKI as a first-line therapy were retrospectively reviewed.

**Results:**

Of the 203 patients enrolled in this study, 139 patients had progression of disease and 63 patients died. The subjects had a median follow up duration of 21.1months and a median PFS of 14.3 months. Partial response (PR) was achieved in 127 (66.1%) patients and stable disease (SD) rate was achieved in 55 (28.6%) patients. In univariate analysis, patients with 2 or higher ECOG score (5.1 *vs* 16 months, *P*=0.033), SD as best overall response (9.5 *vs* 17.9 months, *P*=0.030), extrathoracic metastasis (11.7 *vs* 27.5 months, *P*=0.004), liver metastasis (4.1 *vs* 16.0 months, *P*=0.000), bone metastasis (13.3 *vs* 21.5months, *P*=0.027) and pulmonary embolism (5.5 *vs* 16.6 months, *P*=0.005) had shorter PFS than those without the listed factors. Multivariable *Cox* regression analysis showed best overall response (HR=1.825, 95%CI: 1.107-3.008, *P*=0.018) and liver metastasis (HR=1.694, 95%CI: 1.146-5.756, *P*=0.022) were independent predictive factors of shorter PFS.

**Conclusion:**

Despite the high efficacy of EGFR-TKI, SD as best overall response and liver metastasis predicts poorer PFS in advanced NSCLC patients with *EGFR* gene mutations receiving first-line therapy treatment.

肺癌是目前世界上因肿瘤导致死亡的首要因素^[1]^。近年来，表皮生长因子受体酪氨酸激酶抑制剂（epidermal growth factor receptor tyrosine kinase inhibitor, EGFR-TKI）在针对*EGFR*突变的肺癌治疗中取得了重大进展，带来了肺癌治疗的革命性改变。对于具*EGFR*敏感突变的非小细胞肺癌（non-small cell lung cancer, NSCLC）患者，应用EGFR-TKI治疗可明显延长患者的无进展生存时间，改善预后，且药物相关毒副作用明显低于传统化疗^[2, 3]^。目前，美国国立综合癌症网络（National Comprehensive Cancer Network, NCCN）指南推荐一代EGFR-TKI作为具有*EGFR*基因敏感突变的晚期NSCLC的标准一线治疗^[4]^。然而，具有*EGFR*敏感突变的患者接受一代TKI治疗，仍可出现较大的疗效差异。部分患者应用EGFR-TKI治疗有效后短期即出现耐药，而部分患者却可长期有效。除突变位点的不同外（19外显子缺失突变或21外显子点突变）^[5]^，一些临床特征也可能预测疗效。本研究通过回顾性分析具有*EGFR*敏感突变的晚期NSCLC患者的临床特征，寻找可能的疗效预测因素。

## 1 资料与方法

### 1.1 研究对象

筛选北京协和医院呼吸科肺癌中心数据库中2011年1月1日-2017年12月30日期间，经检测*EGFR*基因突变阳性且曾经接受一代靶向药物一线治疗的门诊患者进行分析。收集其基本信息，治疗过程及随访病历。入选患者需符合以下标准：①年龄≥18岁；②经组织学或细胞学病理确诊为NSCLC；③有足够的组织标本进行*EGFR*基因突变检测，且具有*EGFR*基因19外显子缺失突变或21外显子点突变；④经过完善的影像学评估和分期[包括胸腹计算机断层扫描（computed tomography, CT）、头增强磁共振成像（magnetic resonance imaging, MRI）以及骨扫描]，确定分期Ⅳ期或Ⅲb期不适于接受同步放化疗的患者；⑤一线接受一代EGFR-TKI治疗。本研究经北京协和医院伦理委员会通过，所有患者均签署知情同意书。

### 1.2 方法

收集患者的人口学及临床信息包括性别、发病年龄、影像表现、开始EGFR-TKIs治疗时的东部肿瘤协作（Eastern Cooperative Oncology Group, ECOG）组体能状态评分，吸烟状况（戒烟5年以上的定义为既往吸烟者）及随访资料。依据影像学检查，应用实体瘤疗效评价标准（Response Evaluation Criteria in Solid Tumors, RECIST）1.1进行疗效评估。

### 1.3 统计学方法

应用SPSS 17.0软件进行数据处理和统计分析。*Kaplan-Meier*绘制生存曲线，单因素分析应用四格表χ^2^检验及*Fisher*确切概率，多因素分析采用*Cox*回归分析。双边检验*P* < 0.05为有统计学意义。

## 2 结果

### 2.1 基本状况

研究共入选病例203例，中位年龄为62岁（22岁-79岁），男性患者80例（39.4%），女性患者123例（60.6%）。诊断时大多患者处于疾病晚期，其中Ⅳ期患者191例（94.1%），Ⅲb期患者12例（5.9%）。病理类型以腺癌为主，19外显子缺失突变比例略高于及21外显子点突变，分别为110例（54.2%）及93例（45.8%）（表 1）。

**1:** 患者基本信息 Patient characteristics

Characteristics	Number	Percent (%)
Age (yr), Median (range)	62 (22-79)	
Gender		
Male	80	39.4
Female	123	60.6
Smoking history		
Never smoker	160	78.8
Smoker (Former or current)	30	14.8
Unknown	13	6.4
ECOG score		
0-1	190	93.6
≥2	13	6.4
Stage		
Ⅲb	12	5.9
Ⅳ	191	94.1
Histology		
Adenocarcinoma	196	96.6
Non-adenocarcinoma	7	3.4
*EGFR* mutation		
Exon 19 Del	110	54.2
Exon 21 L858R	93	45.8
EGFR TKI		
Gefitinib	151	74.4
Erlotinib	20	9.9
Icotinib	32	15.8
Best response		
PR	127	66.1
SD	55	28.6
PD	10	5.2
ECOG: Eastern Cooperative Oncology Group; PR: partial response; SD: stable disease; PD: progressive disease.		

### 2.2 治疗反应

截至分析时，发生进展的患者共139例，死亡患者为63例，中位随访时间为21.1个月，中位PFS为14.3个月（95%CI: 11.1-17.5）（图 1）。入选患者共192例可评估疗效，其中最佳疗效为PR的患者为127例（66.1%），SD患者为55例（28.6%），PD患者10例（5.2%）。

**1:**
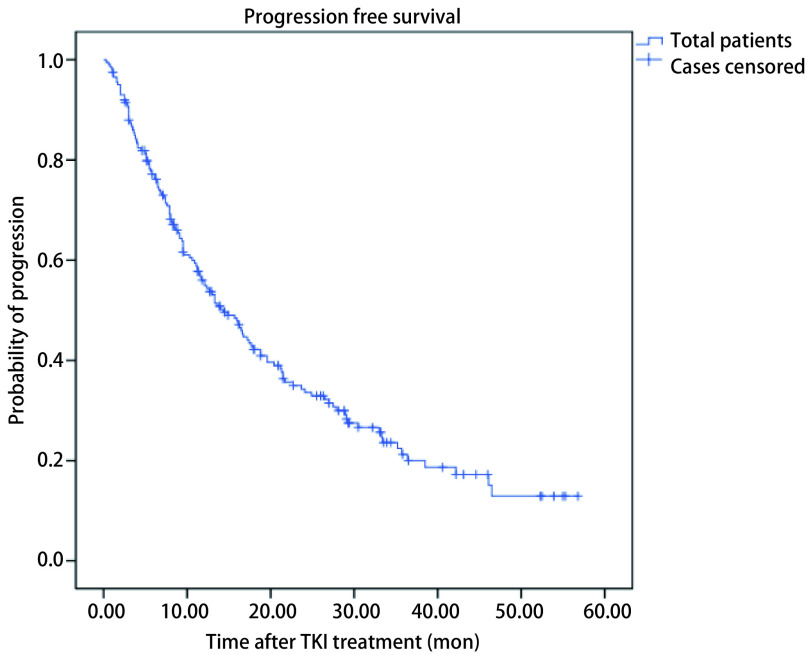
所有患者的无进展生存时间*Kaplan-Meier*曲线 *Kaplan-Meier* curves of PFS of all the patients

### 2.3 无进展生存时间相关的临床单因素分析

单因素分析结果提示，ECOG评分≥2分（5.1个月 *vs* 16个月，*P*=0.033）、最佳疗效为SD（9.5个月 *vs* 17.9个月，*P*=0.030）、合并胸腔外远处转移（11.7个月 *vs* 27.5个月，*P*=0.004）、肝转移（4.1个月 *vs* 16.0个月，*P*=0.000）、骨转移（13.3个月 *vs* 21.5个月，*P*=0.027）和同时并发肺栓塞（5.5个月 *vs* 16.6个月，*P*=0.005）的患者PFS明显缩短，是晚期突变阳性NSCLC患者一线应用TKI治疗的疗效预测因素。同时，年龄 < 65岁患者PFS也相对较短，但并未达到统计学差异。患者的性别、吸烟史、分期、组织学类型、具体突变类型、其他转移部位如脑转移、肾上腺转移、心包转移、均与PFS无明确相关（表 2）。

**2:** 临床因素与无进展生存时间的单因素分析 Univariate analysis of predictive factors associated with PFS

Characteristics	Number	PFS (Mean±SD, mo)	Chi-square	*P*
Age (yr)				
< 65	118	12.1±1.7	3.089	0.079
≥65	85	17.7±2.2		
Gender				
Male	80	11.6±1.0	2.096	0.148
Female	123	17.7±2.3		
Smoking history				
Never smoker	160	16.0±3.6	0.582	0.445
Smoker (Former or current)	30	13.6±1.8		
ECOG score				
0-1	190	16.0±1.6	4.56	0.033
≥2	13	5.1±2.6		
Stage				
Ⅲb	12	20.4±1.6	0.909	0.340
Ⅳ	191	13.3±1.8		
Histology				
Adenocarcinoma	196	15.7±1.7	2.689	0.101
Non-adenocarcinoma	7	5.2±2.1		
*EGFR* mutation				
Exon 19 Del	110	13.6±2.0	1.036	0.309
Exon 21 L858R	93	16.6±4.0		
EGFR TKI				
Gefitinib	151	13±2.2	1.966	0.374
Erlotinib	20	16.0±1.4		
Icotinib	32	27.5±8.3		
Best overall response^*^				
PR	127	17.9±2.0	5.771	0.030
SD	55	9.5±2.7		
Site of metastasis^&^				
Intra thorax	55	27.5±4.8	6.642	0.004
Distance metastasis	136	11.6±1.4		
Liver metastasis^&^				
Yes	22	4.1±1.1	6.507	0.000
No	169	16±1.8		
Bone metastasis^&^				
Yes	70	13.3±2.3	4.887	0.027
No	121	21.5±3.7		
Brain metastasis^&^				
Yes	36	13.6±4.2	0.442	0.506
No	155	17.2±2.8		
Leptomeningeal metastasis				
Yes	12	13±4.5	0.892	0.345
No	179	14.9±1.8		
Adrenal metastasis^&^				
Yes	22	16.0±1.8	0.008	0.930
No	169	16.6±2.9		
Pericardium metastasis^&^				
Yes	11	14.9±7.3	0.148	0.701
No	180	16.6±2.1		
Number of distance metastesis site				
Single	12	14.9±6.7	0.237	0.597
Multiple	124	11.7±1.2		
Pulmonary embolism				
Yes	8	5.5±3.4	8.065	0.005
No	195	16.6±1.8		
^*^ The patients having PD as the initial treatment response were not analyzed; & : Patients with stage Ⅳ disease were included. PFS: progression-free survival.				

### 2.4 无进展生存时间相关的临床多因素分析

将上述相关因素纳入多因素回归分析，结果显示合并肝转移（HR=1.694, 95%CI: 1.146-5.756, *P*=0.022）、最佳治疗反应仅达到SD（HR=1.825, 95%CI: 1.107-3.008, *P*=0.018）是PFS的独立预测因素（表 3）。而ECOG评分高的患者PFS相对较短趋势，但未能达到统计学意义（HR=3.877, 95%CI: 0.869-17.299, *P*=0.076）。其他影响因素胸腔外转移以及合并肺栓塞均未提示为PFS的独立的影响因素。

**3:** 临床因素与无进展生存时间的多因素*Cox*回归分析 Multivariate *Cox* regression analysis of clinical factors affecting the median PFS

	HR	95%CI	*P*
ECOG≥2	3.877	0.869-17.299	0.076
Extrathoracic metastasis	1.220	0.674-2.208	0.512
Liver metastasis	1.694	1.146-5.756	0.022
Bone metastasis	0.724	0.802-2.263	0.259
Best overall response	1.825	1.107-3.008	0.018
Pulmonary embolism	2.519	0.851-1.605	0.337

## 3 讨论

对于有*EGFR*基因敏感突变的患者，应用EGFR-TKIs治疗患者生存时间更长且疾病客观缓解率更高、PFS更长且不良反应更低^[6, 7]^。既往研究表明，除敏感基因外，一些临床因素如ECOG评分、特殊部位的转移（肝、骨、脑）、肿瘤负荷^[8, 9]^等可能与疗效和预后相关，但相关研究病例数相对较少且大多来自非亚裔人群。本研究通过对多个临床因素进行分析，发现最佳疗效和肝转移是*EGFR*突变患者接受EGFR-TKI一线治疗的独立疗效预测因素。

肿瘤缩小程度与PFS和OS的关系早有报道，但结论并不一致。一些研究结果显示肿瘤缩小明显的患者其OS相对更长^[10]^，但也有研究^[11]^表明肿瘤缩小程度与PFS以及OS均无明显相关。然而上述研究中采用的治疗方案以传统化疗为主，客观缓解率较低，与接受靶向治疗的患者存在一定差异。近年来随着靶向治疗应用逐渐广泛，关于靶向治疗疗效相关因素的研究也随之出现。2012年Zhang等报道了^[12]^应用吉非替尼、厄洛替尼等靶向治疗的患者其肿瘤缩小程度与PFS相关。2014年Takeda等^[13]^的研究结果也显示接受靶向治疗后肿瘤的缩小程度和预后相关，其中接受治疗后疗效达PR的患者相对疗效为SD的患者PFS明显延长。但上述研究纳入的患者基因突变状态未明，且并非一线治疗。本研究针对一线治疗患者进行分析，同时对所有可能因素进行多因素分析，结果提示最佳疗效为SD的患者PFS较短，且与PFS独立相关，与既往研究结论相符。提示EGFR-TKI治疗中，最佳疗效是一项独立的预测因素。

肝转移是晚期NSCLC的常见转移部位，在*EGFR*突变的晚期NSCLC中基线肝转移占约20%^[14]^。本研究对肝转移与PFS的关系亦进行了分析，结果显示基线肝转移患者的PFS较无肝转移患者明显缩短，且是一项独立预测因素，与之前研究结论相同^[15, 16]^。肝转移患者PFS较短的机制目前尚不明确，推测可能与以下因素相关：肿瘤细胞激活肝细胞生长因子（hepatocyte growth factor, HGF），而HGF是间质-上皮转化因子（mesenchymal-to-epithelial transition factor, MET）蛋白的配体，可导致MET激活，而MET基因扩增是导致EGFR-TKI耐药机制之一，从而导致耐药发生^[17, 18]^。

此外，体能状况评分既往在多个研究中被认为与晚期NSCLC患者的PFS以及预后相关^[19, 20]^。对于ECOG评分0-1分的患者不论PFS还是生存时间均明显优于评分≥2分的患者。本研究中单因素分析也取得相似结论，但多因素分析结果并未能达到统计学显著性，可能与研究中纳入的ECOG评分≥2分患者相对较少有关。

脑膜转移是*EGFR*基因突变阳性NSCLC的常见转移部位，大多数转移出现在EGFR-TKI治疗中，但部分患者可在诊断初期即合并脑膜转移。由于此类患者常因颅压升高出现多种中枢神经系统症状，且可能存在药物通透性不足，在进行靶向药物治疗同时尚需同时进行放疗及对症治疗^[21]^。本研究对脑膜转移患者进行了分析，结果并未提示两组患者PFS有显著性差异，考虑可能与本组患者合并脑膜转移病例较少相关，尚需要大样本量前瞻性研究进一步分析。

此外，既往研究发现*EGFR*基因19外显子缺失突变和21外显子点突变患者接受一线EGFR-TKI疗效可能存在差异^[22, 23]^，但本研究中两者并未出现疗效的不同，提示这两种突变类型对疗效的影响仍有待于进一步验证。

本研究的不足之处为回顾性研究，患者资料主要来自单一中心的数据库，对于基线病灶以及疗效的评估均来自本中心研究者。多数患者尚存活故未能进行OS相关分析。同时由于检测方法所限，患者*EGFR*突变亚类未能进一步明确及进行分析。其结果有待于大规模前瞻性研究进一步证实。

对于*EGFR*突变阳性的晚期NSCLC患者，一线应用EGFR-TKI治疗效果良好。治疗的最佳疗效及基线肝转移是PFS的独立预测因素。对于存在上述不良因素的患者在临床用药及随访中需要更加关注。
